# Detection of SARS-CoV-2 by real-time PCR under challenging pre-analytical conditions reveals independence of swab media and cooling chain

**DOI:** 10.1038/s41598-021-93028-8

**Published:** 2021-06-30

**Authors:** Sabrina Summer, Ralf Schmidt, Anna Nele Herdina, Isabella Krickl, Julia Madner, Georg Greiner, Florian J. Mayer, Nicole Perkmann-Nagele, Robert Strassl

**Affiliations:** 1 Center for Anatomy and Cell Biology, Medical University of Vienna, 1090 Vienna, Austria; 2 Department of Laboratory Medicine, Division of Clinical Virology, Medical University of Vienna, Waehringer Guertel 18-20, 1090 Vienna, Austria; 3 Department of Gynecological Endocrinology and Reproduction Medicine, Ambulance of In-Vitro Fertilization, Medical University of Vienna, 1090 Vienna, Austria; 4 Ludwig Boltzmann Institute for Hematology and Oncology, Medical University of Vienna, 1090 Vienna, Austria; 5 Ihr Labor, Medical Diagnostic Laboratories, 1220 Vienna, Austria

**Keywords:** Immunology, Microbiology, Molecular biology, Diseases, Health care, Medical research

## Abstract

With global demand for SARS-CoV-2 testing ever rising, shortages in commercially available viral transport media pose a serious problem for laboratories and health care providers. For reliable diagnosis of SARS-CoV-2 and other respiratory viruses, executed by Real-time PCR, the quality of respiratory specimens, predominantly determined by transport and storage conditions, is crucial. Therefore, our aim was to explore the reliability of minimal transport media, comprising saline or the CDC recommended Viral Transport Media (HBSS VTM), for the diagnosis of SARS-CoV-2 and other respiratory viruses (influenza A, respiratory syncytial virus, adenovirus, rhinovirus and human metapneumovirus) compared to commercial products, such as the Universal Transport Media (UTM). We question the assumptions, that the choice of medium and temperature for storage and transport affect the accuracy of viral detection by RT-PCR. Both alternatives to the commercial transport medium (UTM), HBSS VTM or saline, allow adequate detection of SARS-CoV-2 and other respiratory viruses, regardless of storage temperatures up to 28 °C and storage times up to 28 days. Our study revealed the high resilience of SARS-CoV-2 and other respiratory viruses, enabling proper detection in clinical specimens even after long-time storage at high temperatures, independent of the transport medium’s composition.

## Introduction

The rapid spread of the novel coronavirus SARS-CoV-2, probably of zoonotic origin^1^, has led to a continuing COVID-19 pandemic^2^. Extensive laboratory testing for SARS-CoV-2 is currently among the most effective measures to curtail the spread of COVID-19, together with quarantine and social distancing measures^3,4^ as well as vaccination campaigns. Real-time PCR (RT-PCR), typically performed from upper respiratory specimens, represents the current gold standard for SARS-CoV-2 detection. The World Health Organization (WHO) recommends cooled storage (2–8 °C) and the transport of respiratory specimens in a specific viral transport medium (VTM) up to 5 days^4^. Viral transport medium exists in several formulas, all consisting of a buffered salt solution, a complex source of protein and/or amino acids, and antimicrobial agents^5^. The most commonly used commercial transport medium is the universal transport medium (UTM) from Copan Diagnostics which indeed has been proposed for use at room temperature for up to 48 h but not tested for higher temperatures and longer storage times^6^. In the present study, it was used as reference standard. As a more cost-efficient alternative to the commercially available product, the CDC provided a standard operating procedure for the production of viral transport medium suitable for viral detection based on Hanks Balanced Salt Solution, abbreviated as HBSS VTM in this study^7^. Minimal buffered systems, such as saline, have rarely been used for respiratory virus sample collection, transport, and storage in clinical practice in pre-pandemic times.

The COVID-19 pandemic and the associated demand of mass testing have severely challenged worldwide supplies of commercial viral transport media as well as commercial swab kits and reagents for SARS-CoV-2 RT-PCR^5,7^. Due to the increased demand and the shortage in supply, minimal buffered solutions, such as saline, were accessorily used, but their compatibility with viral detection in diagnostics have yet to be assessed. Thus, this study primarily focused on the definition of time- and temperature-dependent alterations detecting SARS-CoV-2 RNA in clinical samples stored (1) in the CDC-provided alternative HBSS VTM^7^, (2) in 0.9% NaCl and (3) in commercially available universal transport media (UTM) at 4 °C to 28 °C for 4 weeks. Additionally, this study aimed to provide evidence on storage and transport conditions suitable for the detection of RNA or DNA, respectively, of other respiratory viruses, including influenza A, respiratory syncytial virus, adenovirus, rhinovirus and human metapneumovirus, for future recommendations.

## Methods

### Sample collection

This study was performed at the Medical University of Vienna, Department of Laboratory Medicine, Division of Clinical Virology (Vienna, Austria, Europe) during the first wave of the pandemic of SARS-CoV-2 in 2020. During this period, nasopharyngeal swabs were collected from patients showing acute respiratory tract infections. The archived respiratory samples were stored in either saline (0.9% NaCl) or UTM (Universal Transport Medium, Cepheid, Copan Diagnostics) for 12–18 h at room temperature until initial patient diagnosis and subsequently frozen at −80 °C.

Stored respiratory specimens from the archive of the Division of Clinical Virology at the Medical University of Vienna, which had been tested positive by routine PCR within the last 2 years for other respiratory viruses (influenza A, respiratory syncytial virus, adenovirus, rhinovirus and human metapneumovirus), were used for comparative evaluation.

### Sample preparation

After one freeze–thaw cycle, 12 anonymized SARS-CoV-2-positive and 3 anonymized SARS-CoV-2-negative respiratory swab samples were diluted 50-fold in 3.5 ml (1) in-house-prepared viral transport medium according to the recipe of the CDC, abbreviated here as HBSS VTM, (500 ml HANKS Balanced Salt Solution HBSS (Gibco), 2% FBS (Gibco), 100 µg/ml Gentamycin (B. Braun Melsungen AG), 0.5 µg/ml Amphotericin B (Cheplapharm))^7^ or (2) saline (0.9% NaCl; B. Braun Melsungen AG), respectively; independent of the medium they were initially stored in. In parallel, 3 archival clinical samples positive for the other respiratory viruses (influenza A virus, adenovirus, respiratory syncytial virus, rhinovirus and human metapneumovirus) and 3 clinical samples negatively tested for the corresponding virus were measured. The samples were prepared as described above.

After the initial measurement at time point “zero” (T0, defined as the first measurement after thawing and dilution), the remaining amount of sample was distributed equally for the storage at 4 °C, 21 °C and 28 °C. T0 represents the initial measurement after the sample preparation. 700 µl (500 µl + 200 µl residual volume required by extraction system) of the pool were used for RNA extraction at each specified time point. The samples were stored at the different temperatures (4 °C, 21 °C and 28 °C) for up to 28 days. Total RNA was extracted after 4 (T4), 7 (T7), 14 (T14) and 28 (T28) days. pH values, which served as a surrogate parameter for microbial contamination, were determined before each measurement.

### RNA isolation and RT-PCR analysis

Total RNA of SARS-CoV-2 and the other respiratory viruses was extracted from 500 µl of the samples with the AltoStar Automation System AM16 (Altona Diagnostics, Hamburg, Germany) according to the manufacturer’s instructions and eluted in 45 µl elution buffer (Altona Diagnostics, Hamburg, Germany). RT-PCR was carried out using the RealStar SARS-CoV-2 RT-PCR kit (Altona Diagnostics, Hamburg, Germany) targeting the SARS-CoV-2-specific S gene and the betacoronavirus-specific E gene on a Bio-Rad CFX Connect Real-Time PCR Detection System (Bio-Rad Laboratories, Hercules, California, USA). Cq-values (quantification cycle values; cycle threshold values as calculated specifically by the Bio-Rad CFX Manager software, as defined by the MIQE guidelines described in Bustin et al.^8^) less than 45 cycles for both tested targets were interpreted as positive for SARS-CoV-2. Positive specimens were further divided into SARS-CoV-2++ (Cq < 27) and SARS-CoV-2 + (Cq ≥ 27). Internal controls were used to confirm measurement integrity.

The presence of other respiratory viruses used in this study, influenza A virus, adenovirus, respiratory syncytial virus, rhinovirus and human metapneumovirus, were confirmed by TaqMan-based RT-PCR using the LightCycler Multiplex RNA Virus Master (Roche Diagnostics, Rotkreuz, Switzerland) on a Bio-Rad CFX Connect Real-Time PCR Detection System (Bio-Rad Laboratories, Hercules, California, USA). RT-PCR was performed using the routine testing setup for respiratory viruses at the Medical University of Vienna, Department of Laboratory Medicine, Division of Clinical Virology by the following cycling protocol: 8 min at 53 °C, 30 s 95 °C, followed by 50 cycles of 1 s at 95 °C, 40 s at 60 °C, 1 s at 72 °C and 30 s at 40 °C. Primers and probes^9–13^ are listed in Table 1.

**Table 1:** RT-PCR primers and probes used in this study.

Respiratory virus	Forward primer	Reverse primer	Probe
Influenza A virus	AAGACCAATYCTGTCACCTCTGA	CAAAGCGTCTACGCTGCAGTCC	FAM-5′-TTTGTKTTCACGCTCACCGT-3′-TAMRA
Adenovirus	GCCACGGTGGGGTTTCTAAACTT	GCCCCAGTGGTCTTACATGCACATC	FAM-5′-TGCACCAGACCCGGGCTCAGGTACTCCGA-3′-TAMRA
Respiratory syncytial virus	GGCAAATATGGAAACATACGTGAA	TCTTTTTCTAGGACATTGTAYTGAACAG	FAM-5′- CTGTGTATGTGGAGCCTTCGTGAAGCT-3′-TAMRA
Rhinovirus	CY + AGCC + TGCGTGGC	GAAACACGGACACCCAAAGTA	FAM-5′-TCCTCCGGCCCCTGAATGYGGC-3′-BBQ
Human metapneumovirus	CATATAAGCATGCTATATTAAAAGAGTCTC	CCTATTTCTGCAGCATATTTGTAATCAG	FAM-5′-TGYAATGATGAGGGTGTCACTGCGGTTG-3′-TAMRA

### Data analysis

Cq-values (quantification cycle values: Ct or cycle threshold values defined according to the MIQE guidelines described in Bustin et al.^8^) were exported from CFX Manager Dx Software version 3.1. ΔCq-values were calculated in Microsoft Excel Version 2019 as the differences between the target Cq measured at the dedicated day and temperature and the target Cq of the initial measurement after thawing (T0). “Not detectable” signals (N/A) were set to 45 cycles for analysis (total number of cycles of the RT-PCR).

Details on sample sizes are provided in the figure legends. Statistical analysis and calculating the mean Cq-values were done in GraphPad Prism Version 5.0 (GraphPad). Kolmogorov–Smirnov normality test was done in GraphPad Prism Version 5.0 (GraphPad). In most cases, a two-tailed repeated measurement ANOVA was performed with adjustments for multiple comparisons, following the Bonferroni post-hoc test when indicated. When data was not normally distributed, a Wilcoxon signed-rank test was done. Significant differences *(p* < 0.05) were marked by a * above the corresponding curves in the figures.

## Results

### HBSS VTM or saline are adequate alternatives to UTM as transport media for SARS-CoV-2 specimens in molecular diagnostics

The thermal long-time stability of the viral RNA is a key aspect in diagnostics assessed by RT-PCR. Thus, we tested swab specimens of 12 positively-tested SARS-CoV-2 patients which were diluted in viral transport medium prepared by the authors according to the CDC recommendation (HBSS VTM) or saline (0.9% NaCl), respectively, and stored over 28 days at 4 °C, 21 °C and 28 °C. Additionally, 6 undiluted swab specimens stored in Universal Transport Medium from Cepheid, Copan Diagnostics (UTM) and 6 undiluted specimens stored in saline were stored at 21 °C for 14 days, to evaluate a possible impact of the dilution factor on the results.

Firstly, we assessed the effect of the temperature and storage time on RNA stability under consideration of the different medium background. For each transport medium, we analyzed a range of SARS-CoV-2 specimens with original Cq- values from approximately 13 to 32. Three negatively tested specimens were included as control. As quality control we calculated the Cq-values of the positive controls which shows a mean Cq of 28.65 ± 0.12 for the E-gene and 29.25 ± 0.49 for the S-gene.

**Figure 1:**
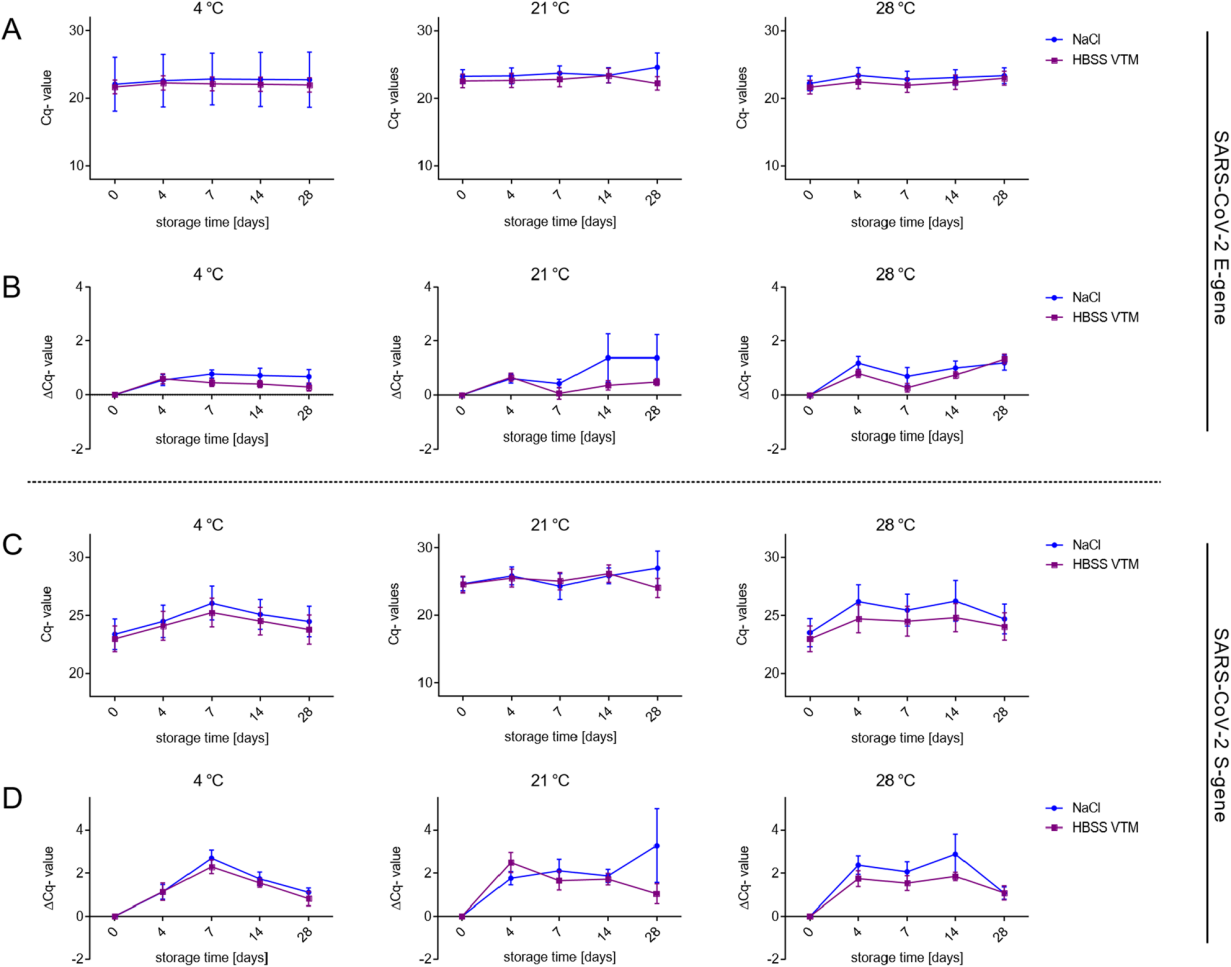
HBSS VTM and saline are both suitable transport media for SARS-CoV-2 specimens at storage temperatures up to 28 °C. RT-PCR measurements of clinical specimens positive for SARS-CoV-2 stored in NaCl (blue point) or HBSS VTM medium (purple rectangle) over 4 weeks at 3 different temperatures (4 °C, 21 °C and 28 °C). ΔCq-values ± SEM for SARS-CoV-2 at 4 °C, 21 °C and 28 °C *n* = 12 for NaCl and HBSS VTM, respectively, are shown. *p-values < 0.05, repeated measurement ANOVA.

There was no apparent difference detectable in the Cq-values of specimens stored in HBSS VTM or saline (Fig. 1 and Supplementary Dataset). Even at higher storage temperatures (21 °C and 28 °C) the viral RNA was clearly detectable by RT-PCR, showing no significant drop in RNA stability over time. However, higher temperatures seem to result in an increased variation in the data after longer storage, especially for the S-gene (mean ΔCq NaCl 17.953 ± 3.51; mean ΔCq HBSS VTM 16.001 ± 2.891) and the E-gene (mean ΔCq NaCl 13.441 ± 2.915; mean ΔCq HBSS VTM 12.136 ± 2.476) at 21 °C (Fig. 1).

**Figure 2:**
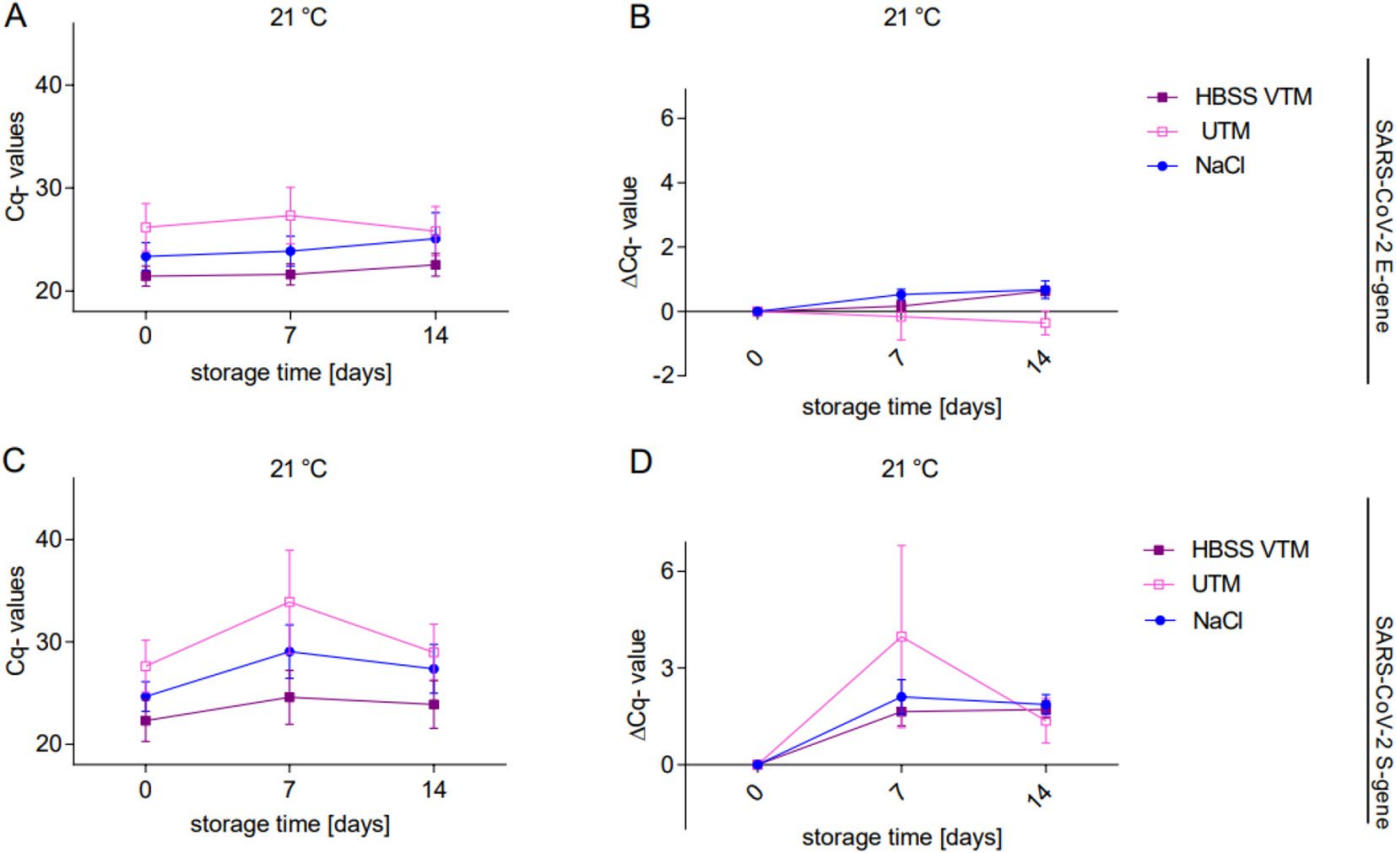
HBSS VTM and saline are both suitable alternatives to UTM as transport media for SARS-CoV-2 specimens. RT-PCR measurements of clinical specimens positive for SARS-CoV-2 stored in NaCl (blue point), HBSS VTM (purple rectangle full) or UTM medium (rose rectangle empty) over 14 days at 21 °C. ΔCq-values ± SEM for NaCl or HBSS VTM *n* = 16 (12 from Fig. 1 and 6 additional specimens in parallel to 6 in UTM) and UTM *n* = 6 are shown. *p-values < 0.05, repeated measurement ANOVA. In all groups the viral RNA levels were adequately stable for RT-PCR detection, independent of the storage conditions.

**Figure 3:**
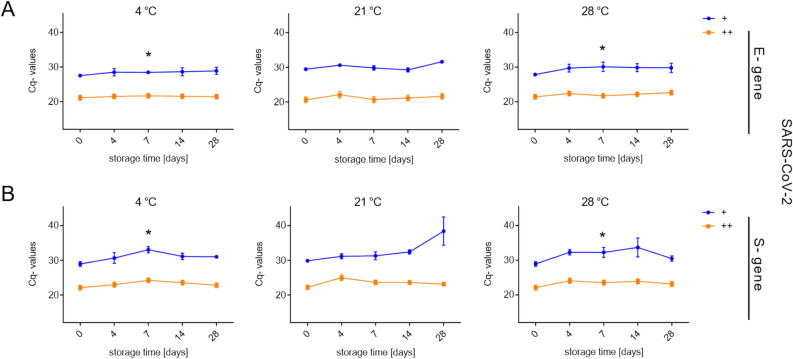
Long-term storage, even at high temperatures, allows adequate detection of weakly positive SARS-CoV-2 specimens. RT-PCR measurements of clinical specimens strongly (orange rectangle) and weakly positive (blue point) for SARS-CoV-2 stored in NaCl or HBSS VTM over 4 weeks at 3 different temperatures (4 °C, 21 °C and 28 °C). Cq-values ± SEM for SARS-CoV-2 + at 4 °C *n* = 4; at 21 °C *n* = 10 and 28 °C *n* = 2 and for SARS-CoV-2++ at 4 °C *n* = 20; at 21 °C *n* = 17 and 28 °C *n* = 22 are shown. *p-values < 0.05, repeated measurement ANOVA and Wilcoxon signed rank test. The stability of SARS-CoV-2 + samples kept at 4 °C and 28 °C varied significantly from SARS-CoV-2++ after 7 days (* 4 °C *p*_E_ = 0.0008, *p*_S_ =  < 0.0001; 28 °C *p*_E_ = 0.0049, *p*_S_ = 0.0001); however, no significant changes in the Cq-values could be observed for the specimens stored at 21 °C (*p*_E_ = 0.73, *p*_S_ = 0.60).

Closer evaluation of the thermal stability of specimens stored at 21 °C in HBSS VTM or saline for 14 days revealed no significant variation between the measurements (Fig. 2), in contrast to our previous observations (Fig. 1). However, specimens stored in UTM showed a trend towards higher variations, especially at day 7 (Fig. 2). Comparing the two tested alternative media, HBSS VTM or saline, with the commercially available UTM, no significant differences in the ΔCq-values could be observed over time (mean ΔCq E T14 for UTM −0.3583 ± 0.3683, HBSS VTM 0.6344 ± 0.1524, NaCl 0.6753 ± 0.2770, *p* = 0.101, mean ΔCq S T14 for UTM 1.365 ± 0.6909, HBSS VTM 1.716 ± 0.2671, NaCl 1.867 ± 0.3079, *p* = 0.637; Fig. 2). However, specimens stored in saline at 21 °C showed on trend an increase in the ΔCq after 7 days of storage. Besides, the initial viral load did not affect RNA stability either. We analyzed a range of SARS-CoV-2++ specimens with Cq < 27 (mean Cq 22.16 ± 0.62) to SARS-CoV-2 + specimens with Cq ≥ 27 (mean Cq 29.86 ± 0.48), independent of the medium they were stored in (Fig. 3). No differences could be observed in the detection of the two target genes E and S in specimens characterized by low and high viral load, respectively, considering the different medium background and time and temperature of storage (Fig. 3).

Storage in a medium composed specifically for virus transport (either HBSS VTM or UTM) favors microbial contamination, not observed in specimens stored in saline (Supplementary Figure S1); however, this did not impair the analysis. Microbial contamination was defined here by the macroscopic formation of white sediment and drop in pH-value.

In general, the SARS-CoV-2 RNA could be consistently detected and analysed by RT-PCR, independent of the media and the temperature (tested up to 28 °C) they were stored in (Fig. 1 and Supplementary Dataset). However, further analysis suggests slight differences in the stability of the target genes to diagnose SARS-CoV-2 (S and E gene). Both genes seem to be on-trend more stable at 4 °C and 28 °C (Fig. 1 and Supplementary Dataset); notably, no significant changes could be observed between the different transport media. The S gene differs strongly in its stability depending on the storage medium after 14 and up to 28 days; with some specimens showing an increased drop in Cq-values at day 28 (Fig. 1 and Supplementary Dataset).

### High stability of other respiratory viruses in HBSS VTM and saline at higher temperatures

**Figure 4:**
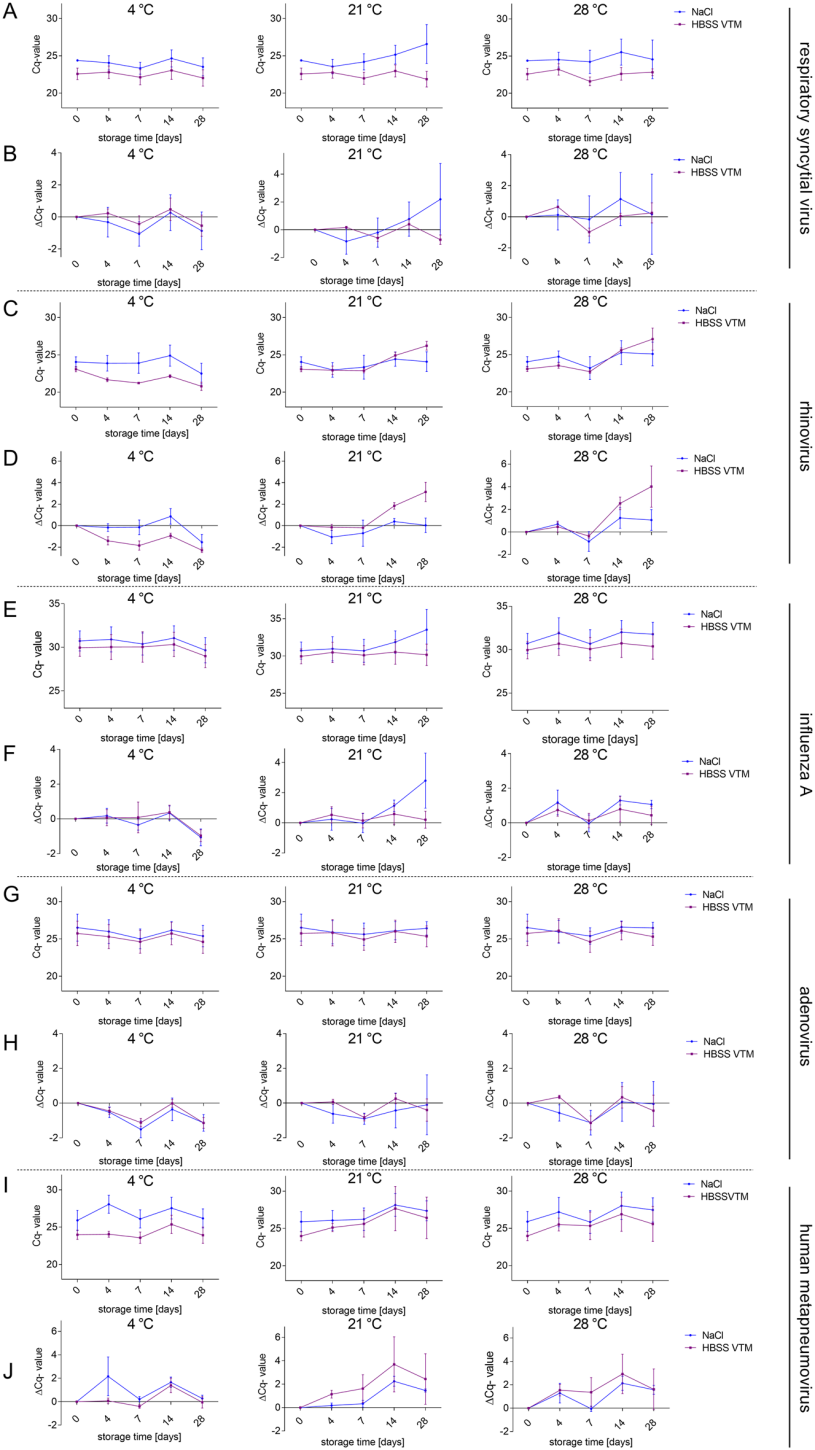
HBSS VTM and saline are both suitable transport media for respiratory virus specimens at storage temperatures up to 28 °C. RT-PCR measurements of clinical specimens positive for, respiratory syncytial virus (**A**,**B**), rhinovirus (**C**,**D**), influenza A (**E**,**F**), adenovirus (**G**,**H**) and human metapneumovirus (**I**,**J**) stored in NaCl (blue point) or HBSS VTM medium (purple rectangle) over 4 weeks at 3 different temperatures (4 °C, 21 °C and 28 °C). *n* = 3 for each group are shown. *p-values < 0.05, repeated measurement ANOVA.

Previous studies have already discussed the high resilience of SARS-CoV-2 to higher temperatures compared to other respiratory viruses like influenza^14,15^. In this study we re-evaluated the thermal stability of influenza A and other respiratory viruses, including adenovirus, respiratory syncytial virus, rhinovirus and human metapneumovirus, for their detection by RT-PCR using alternative minimal media for storage. Three swabs collected from clinical patients positively tested for influenza A (mean Cq 30.34 ± 0.70), respiratory syncytial virus (mean Cq 23.49 ± 0.53), adenovirus (mean Cq 26.13 ± 1.10), rhinovirus (mean Cq 23.56 ± 0.40) and human metapneumovirus (mean Cq 24.94 ± 0.79), respectively, were stored in HBSS VTM or saline over 4 weeks at constant 4 °C, 21 °C and 28 °C, respectively (Fig. 4). The detection of the respiratory viruses was stable, even at higher temperatures of 28 °C over the observation time; however, respiratory syncytial virus, rhinovirus and influenza A stored at 21 °C showed a destabilization of the detected target gene, mainly when stored in saline (Fig. 4A–F). In general, the Cq-values of influenza A, respiratory syncytial virus, adenovirus, rhinovirus and human metapneumovirus stored in HBSS VTM or saline were consistently detectable and showed no significant drop in RNA stability over time (Fig. 4 and Supplementary Dataset). Thus, in contrast to previous studies proposing a temperature-dependent instability of influenza and possible other respiratory viruses^16,17^, our data suggest their genetic material to be highly stable for diagnostics by RT-PCR.

## Discussion

The COVID-19 pandemic has re-emphasized the importance of molecular diagnostics for the acquisition of timely results aiding in the containment of the pathogen. In the case of SARS-CoV-2, previous studies have shown that the virus, in comparison to other respiratory viruses, like influenza, is more resilient to higher temperatures^14,15^. Future demand for mass testing, requiring complex logistics with long transports, required a study determining the thermostability of the virus over a longer time period. Higher outdoor temperatures or storage at non-cooled areas combined with a long transport time of respiratory specimens could affect the virus detection capabilities of molecular methods such as RT-PCR. This could especially be a problem for mass testing and testing in countries with poor infrastructure where rapid, cooled transport and the availability of commercial transport media for respiratory specimens cannot be guaranteed. Besides, the extensive usage of swab tests and the limited access to commercial products emphasize the need for alternatives.

### Thermostability up to 28 °C

In the present study, we evaluated the stability of SARS-CoV-2 and a variety of other respiratory viruses, representing different families varying in size, structure and genetic material, for diagnostic analysis by RT-PCR at temperatures up to 28 °C, considering possible effects mediated by the transport medium. In general, it is assumed that the sensitivity of viruses to heat increases with size and the existence of an envelope; hence, non-enveloped viruses are the most stable. However, there are only a few studies reporting thermostability of structurally different respiratory viruses under identical conditions^17,18^. In our study, we could not detect a correlation between structure and stability. Comparing the enveloped SARS-CoV-2 virus with non-enveloped respiratory viruses (rhinovirus and adenovirus), we detected no significant differences in the long-term thermostability. RNA or DNA, respectively, of all viral species observed in this study showed a high thermal stability.

### Long-term stability up to 28 days

Using positively tested clinical specimens, we demonstrated the remarkable stability of the viral RNA upon long-time storage (up to 28 days) at higher temperatures (21 °C and 28 °C), independent of the transport medium (Fig. 1). Storage in alternatives to the commercially available UTM medium, such as the CDC recommended viral transport medium (HBSS VTM) or saline, were equally suitable for adequate detection of the two target genes E (betacoronavirus-specific) and S (SARS-CoV-2-specific) in specimens characterized by a low and high viral load, respectively (Fig. 3). However, at higher temperatures (up to 28 °C), samples stored in both self-made HBSS VTM or commercial UTM proved to be more susceptible to microbial contamination, even when supplemented with antibiotics. Nevertheless, occurrent contamination has neither affected sample preparation nor target detection.

### Merits of the present study

The relatively high resilience of viral RNA towards higher temperatures has been previously reported for some respiratory viruses. Our observations confirmed this for a variety of respiratory viruses, including SARS-CoV-2^16,20–23^, however, our study presented additional data on “time” as an essential factor potentially affecting viral detection. We also showed, that viral specimens stored in UTM remain stable for RNA detection at temperatures up to 28 °C and for storage times of at least 14 days (the maximum tested for this medium in the present study). This expands the stability range of 48 h at room temperature given in the product information by Copan Diagnostics considerably.

This study mainly focused on two alternative transport solutions, namely HBSS VTM and saline. The evaluation of other in-house recipes for viral transport media, or dry swabs was outside the scope of the present study, as was the evaluation of swab types for sample collection. Furthermore, some conditions tested are underrepresented in terms of sample size; thus, to some extent the data might be subject to biases. Next to the definition of storage medium, time and temperature, it seems that the sampling procedure might be the critical factor to ensure efficient routine testing. Here, the UTM kits might have an advantage due to included glass beads. Whether these can facilitate the release of the virus particles from the swab, was not tested in the scope of the present study. Notably, in this study only temperatures up to 28 °C were tested. The authors do not exclude that even higher temperatures would destabilize the virus, and consequently, the viral RNA; as already discussed in current literature^19^. Future studies will address these aspects.

## Conclusions

For the handling of SARS-CoV-2 and other respiratory virus specimens in diagnostics, the WHO recommends a maximal storage of 5 days at 2–8 °C^4^. Our findings that even a minimal buffered system such as saline provides a suitable viral transport medium for stable long-time storage of at least 28 days of the clinical samples, lacking significant changes in the detection levels of the target genes, not even in case of temperatures up to 28 °C, could be advantageous to increase future testing capacities. In regards to minimal but not significant alterations in the detected signal, SARS-CoV-2 RNA is highly resilient to temperatures up to 28 °C, enabling a sufficiently robust detection of SARS-CoV-2 by RT-PCR for diagnostics.

In summary, we have shown that the stability of SARS-CoV-2 RNA in human respiratory swab specimens seems to be robust (Fig. 1)^19^. Increasing temperatures and long-term storage conditions did not affect its stability. Additionally, our data confirmed that even minimal transport media such as saline and in-house prepared transport media (HBSS VTM), which are cheaper and more easily accessible, provide adequate alternatives to the commercially available UTM. Both can be deployed for transporting, and even for long-term preservation of SARS-CoV-2 specimens at higher outdoor temperatures (up to 28 °C) or non-cooled transport vehicles. Our data on the thermal RNA or DNA stability of respiratory viruses from different families will help assessing the likelihood of viral stability through long, non-cooled transports or sample storages and it emphasizes that an increased capacity of testing for widespread screening of SARS-CoV-2 and early diagnosis of COVID-19 can be achieved.

### Statement of ethical approval

The local Institutional Review Board deemed the study exempt from review. Respiratory swab samples from patients with suspected SARS-CoV-2 infection were first assessed at the Division of Clinical Virology at the Department of Laboratory Medicine at the Medical University of Vienna. A collection of left-over material of positive samples was anonymized and then randomized for purposes of this study before stability analysis. Thus, in correspondence with representatives of the ethical board of the Medical University of Vienna, no separate consultation of the ethics committee was necessary to guarantee ethical standards for conducting biomedical research as required by the Declaration of Helsinki.

## Supplementary Information

Supplementary Information 1.

Supplementary Information 2.

## Data Availability

The datasets generated and analyzed during the current study are included in this article (and its Supplementary Information files).
