# Supplementary material for: Detection of SARS-CoV-2 by real-time PCR under challenging pre-analytical conditions reveals independence of swab media and cooling chain
Source: Sci Rep. 2021 Jun 30;11:13592. doi: 10.1038/s41598-021-93028-8 (PMC8245405; doi:10.1038/s41598-021-93028-8)
Supplement: Supplementary file 1 — Supplementary Information 1. [file 41598_2021_93028_MOESM1_ESM.docx]

**Supplementary Information for**

**Detection of SARS-CoV-2 by Real-Time PCR under challenging pre-analytical conditions reveals independence of swab media and cooling chain**

Sabrina Summer^1,†^, Ralf Schmidt^2,†^, Anna Nele Herdina^2^, Isabella Krickl^3^, Julia Madner^3^, Georg Greine^2,4,5^, Florian J. Mayer^2^, Nicole Perkmann-Nagele^2^, Robert Strassl^[2](https://orcid.org/0000-0002-2157-0382)2), *^

^†^Joint first authors who contributed equally to the work.

*corresponding author: [robert.strassl@meduniwien.ac.at](mailto:robert.strassl@meduniwien.ac.at)

^1^Center for Anatomy & Cell Biology, Medical University of Vienna, Vienna, A-1090, Austria

^2^Department of Laboratory Medicine, Division of Clinical Virology, Medical University of Vienna, Vienna, A-1090, Austria

^3^Department of Gynecological Endocrinology and Reproduction Medicine, Ambulance of In-Vitro Fertilization, Medical University of Vienna, Vienna, A-1090, Austria

^4^Ludwig Boltzmann Institute for Hematology and Oncology, Medical University of Vienna, Vienna, A-1090, Austria

^5^Ihr Labor, Medical Diagnostic Laboratories, Vienna, A-1220, Austria

**Supplementary Figures**

Supplementary Figure S1


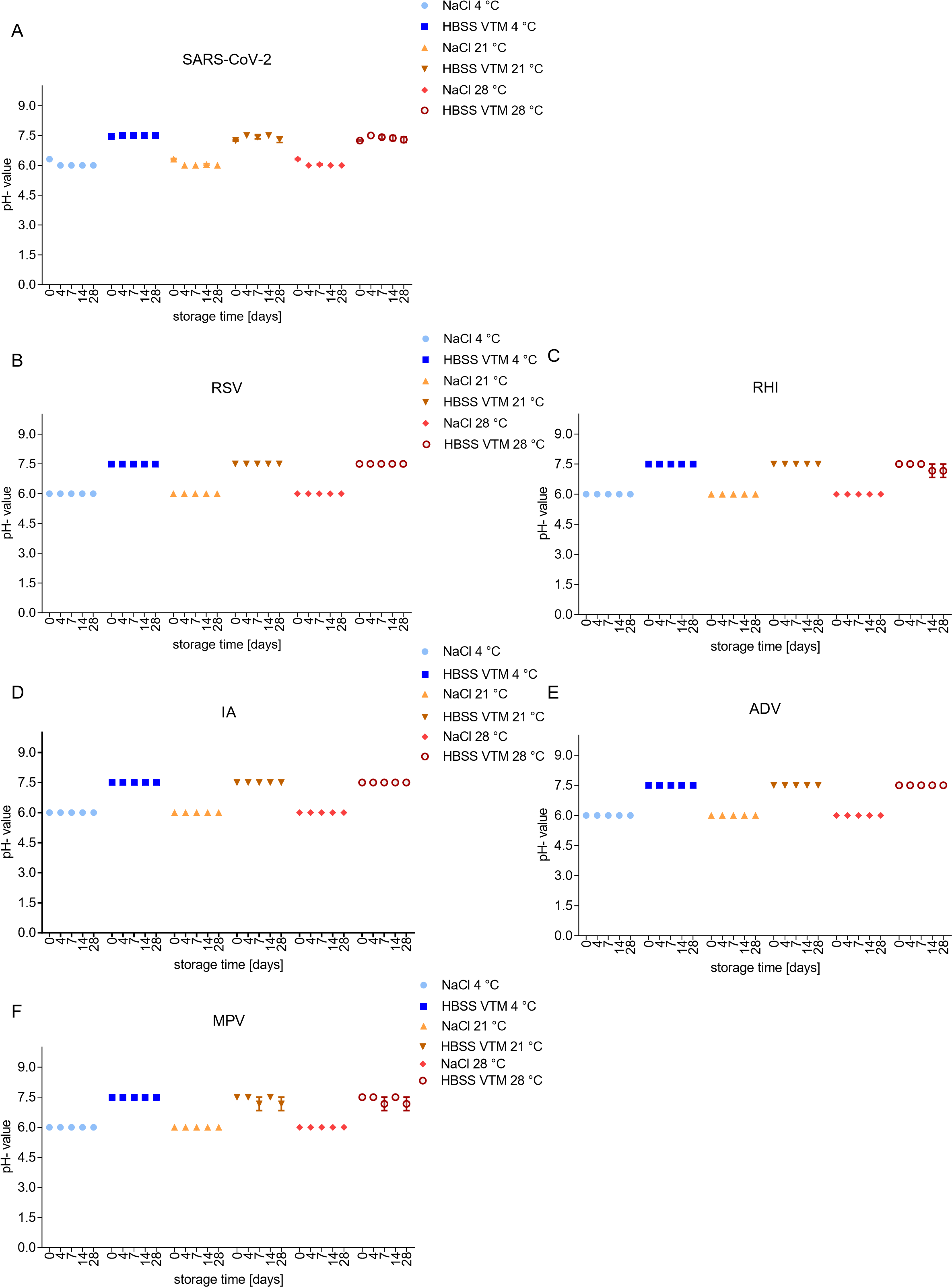


**Supplementary Figure S1: pH-values tracked during storage of SARS-CoV-2 specimens in NaCl and HBSS VTM over 28 days at 4 °C, 21 °C and 28 °C.**

pH-values determined for clinical specimens positive for SARS-CoV-2 (A) and other respiratory viruses (respiratory syncytial virus (B), rhinovirus (C), influenza A (D), adenovirus (E) and human metapneumovirus (F)) stored in NaCl or HBSS VTM medium over 28 days at three different temperatures (4 °C, 21 °C and 28 °C). pH was determioned before each measurement.
